# Comparative evaluation of antimicrobial efficacy of 3C antibiotic paste with triple antibiotic paste as root canal filling material for primary teeth: An in-vitro study

**DOI:** 10.12688/f1000research.133458.1

**Published:** 2023-06-27

**Authors:** Aakriti Chandra, Nilima Thosar

**Affiliations:** 1 Department of Pediatric and Preventive Dentistry, Sharad Pawar Dental College and Hospital, Datta Meghe Institute of Higher Education and Research (Deemed to be University), Sawangi Meghe, Wardha, Maharastra, 442001, India

**Keywords:** Triple Antibiotic Paste, Clindamycin, Cefaclor, root canal filling material

## Abstract

Root canal infection in primary teeth is polymicrobial in nature. Most resistant micro-organisms, such as *E.faecalis* survive in chronic infection of the root canal at the periapical area and are difficult to remove by various root canal irrigants. *C.albicans* has been found in chronic infections of root canals of primary teeth due to its ability to invade dentinal tubules. The multiple bacteria strains which cause endodontic infections, make it difficult to clean root canals with a single effective antibiotic. Hence Triple Antibiotic Paste (TAP) has been previously introduced, consisting of ciprofloxacin, metronidazole, and minocycline. Although this mixture aids in pulp regeneration and has been used to disinfect root canal systems, it is linked to tooth discolouration. The aim of this study is to evaluate and compare the antimicrobial efficacy of a newly formulated 3C antibiotic paste (consisting of Ciprofloxacin, Clindamycin, and Cefaclor) with conventional TAP against *E. faecalis* and *C. albicans.* In this in-vitro laboratory study, pure culture of *C. albicans* and *E. faecalis* will be grown on Sabouraud’s dextrose agar (SD) agar and Brain heart infusion agar (BHI) agar respectively and will be suspended for 24 hours at 37°C. For comparison of antimicrobial efficacy, the zone of inhibition will be for the 3C antibiotic paste against conventional TAP (control) for *E. faecalis* and *C. albicans*, and will be determined using digital calliper in millimetre after every 24 and 72 hours. We hypothesise that the newly formulated 3C paste will have better antimicrobial efficacy when compared with conventional TAP. It is expected that a newly formulated 3C paste will prove to be the successful as root canal filling material for primary teeth.

## Introduction

### Background and rationale

Microorganisms are crucial in the development of pulpal and periapical diseases. The polymicrobial character of the infection in the root canal system is primarily dominated by anaerobes. ^1^ One of the frequently isolated among them is *E. faecalis.* *E. faecalis* is one of the most resistant micro-organisms and is most difficult to remove from the periapical areas of the tooth through conventional methods. ^2^ About 23–70% *E. faecalis* can be isolated from infected root canals with symptoms of chronic apical periodontitis. ^3^

Among Fungi, Candida species are found in about 3–18% of infected root canals. ^4^

According to earlier research, *C. albicans* and *E. faecalis* can infiltrate the dentinal tubules to varying depths, hence removal of these bacteria is facilitated by the use of an efficient antimicrobial agent in the root canal. ^5^

Over the years, several materials were tested in an effort to find an ideal obturating material for a primary tooth, such as zinc oxide eugenol, Maisto paste, Triple Antibiotic Paste etc. ^6^ The antimicrobial efficacy of Triple Antibiotic Paste was evaluated for its use as root canal filling material with different modifications by various authors. ^7^^,^^8^

In Triple Antibiotic Paste, the antibiotics used are: Ciprofloxacin, metronidazole, and minocycline in 1:1:1 concentration. ^9^

Ciprofloxacin is a second-generation fluoroquinolone antibiotics active against a broad range of bacteria, especially *Enterobacteriaceae* and *Neisseria.* ^10^ Metronidazole is a nitro imidazole compound having a broad spectrum of anti-anaerobic and anti-protozoal activity. ^9^ Minocycline is a member of the Tetracycline class of broad-spectrum antibiotics, which work by exerting bacteriostatic action on a wide variety of microorganisms. ^8^^,^^9^

But, Minocycline causes discoloration of tooth as stated by various authors. ^11^^,^^12^ For the same reason, Sara Alsubait *et al* (2020) in their study had replaced minocycline with Cefaclor. Cefaclor is a second generation cephalosporin antibiotic which is mostly effective against most strains of Gram positive aerobes as well as gram negative aerobes. ^10^

A broad-spectrum antibiotic called Clindamycin is effective against infections that produce beta-lactamases as well as against aerobic and anaerobic microorganisms. ^10^ Prasanna *et al* (2020) in their study concluded that Clindamycin could inhibit the levels of *C albicans* by 90% but Metronidazole could not inhibit the *C. albicans.* ^8^

With respect to above literature, considering the disadvantage associated with Minocycline and reduced antimicrobial efficacy of Metronidazole, the present study has planned to use the 3C paste consisting of Ciprofloxacin, Clindamycin, and Cefaclor as a root canal filling material for primary teeth.

The aim of the present study will be to evaluate and compare the antimicrobial efficacy of 3C paste with conventional Triple Antibiotic Paste against *E. faecalis* and *C. albicans.*

### Objectives

The objectives of the study will be to evaluate the antimicrobial efficacy of 3C paste and triple antibiotic paste against *E. faecalis* and *C. albicans*; and also to compare the antimicrobial efficacy of 3C paste with Triple Antibiotic Paste against *E. Faecalis* and *C. albicans.*

## Protocol

### Study design

This *in vitro* study will be conducted at Department of Pediatric and Preventive Dentistry, Sharad Pawar Dental College and Hospital, Datta Meghe Institute of Higher Education and Research, Sawangi Meghe, Wardha in collaboration with the Institution of Pharmaceutical Education and Research, Borgaon (Meghe), Wardha. The present study will be an *in vitro* experimental microbiological study.

### Ethics statement

Although no human or animal participants will be involved in this research, as per institutional guidelines, ethical approval for the study was obtained from the institutional ethics committee of Datta Meghe Institute of Higher Education and Research, Wardha (Ref. No. DMIHER (DU)/IEC/2023/564 approved on 02/02/2023).

### Methods

For the antimicrobial analysis brain heart infusion agar (BHI), Sabouraud’s dextrose agar (SD), Agar plates, Petri dishes *, E. faecalis* strain *, C.albicans* strain will be used. For preparation of paste, Ciprofloxacin, Clindamycin, Cefaclor, Metronidazole, Minocycline, Propylene glycol/macrogol will be required. These steps are outlined in full in the sections below.

*E. faecalis* strain and *C.albicans* strain and the powder form of antibiotics will be included for the study. However other bacterial strains will be excluded from the study.

*Preparation of Triple Antibiotic Paste*

Triple Antibiotic Paste will be prepared by mixing equal proportion of Ciprofloxacin, Metronidazole, and Minocycline (1:1:1). Then a part of the solvent (propylene glycol- macrogol) in 1:1 ratio will be dispensed to obtain soft, uniform consistency. The resultant paste will be stored in an airtight container.

*Preparation of 3C antibiotic paste*

3C paste will be prepared in the same manner as Triple Antibiotic Paste. In 3C paste, the antibiotics Ciprofloxacin, Cefaclor, and Clindamycin (1:1:1) will be mixed with one part of Propylene Glycol-one part of Macrogol in 5:1 (powder: liquid) ratio to get desired consistency as required for the paste.

*Procedure*

Pure colonies of *C. albicans* ( ATCC 2091) and *E. faecalis* ( ATCC 35550) will be grown on Sabouraud’s dextrose (SD) agar and Brain heart infusion (BHI) agar respectively and will be suspended in 5 ml of SD broth and BHI broth for 24 hours at 37°C.

The actively growing microorganisms in broth cultures will have turbidity adjusted to the 0.5 McFarland standard. ^3^^,^^5^

The agar well diffusion method will then be followed. Wells of 3-4 mm will be prepared in the respective agar and then all the wells will be loaded with test material. The agar plates will be incubated at 37°C for 24 and 72 hours to achieve good bacterial growth before analysis. The inhibition zones of microbes will be evaluated using digital calliper in millimetre after every 24 and 72 hours. All the procedures will be repeated in triplicates to minimize errors.

*Primary outcomes to be measured*

The antimicrobial efficacy of newly formulated 3C antibiotic paste will be measured by bacterial growth and will be compared with the Triple Antibiotic Paste.

*Data analysis and statistical plan*

Statistical analysis will be done using software SPSS Version 23.0. P value will be considered significant for <0.05. Descriptive analysis, i.e., mean distribution of inhibition zones will be recorded. A repeated measures ANOVA for intragroup comparison will be done on application of triplicate test of every group. Independent sample t test will be done for intergroup comparison.

*Expected outcome*

It is expected that a newly formulated 3C paste will be able to show antimicrobial efficacy and will prove to be the successful as root canal filling material for primary teeth.

*Dissemination*

This study will be published in an indexed journal.

### Study status

Not yet started.

## Discussion

Complete eradication of micro-organism from root canal space is a challenging task in primary teeth due to morphology of the root canal. The multiple bacteria strains which cause endodontic infections, make it impossible to clean root canals with a single effective antimicrobial agent. ^9^ Hence, a combination of drugs commonly known as Triple Antibiotic Paste (TAP) was introduced as treatment modality by Hoshino *et al* (1996) to eliminate the micro-organisms from infected root canal of primary teeth. It is usually composed of Ciprofloxacin, metronidazole, and minocycline (1:1:1). This mixture aids in pulp regeneration as well as the disinfection of the root canal system. ^13^

An *in vivo* study conducted by Pinky C *et al* (2011) using two different combination of antibiotics in deciduous teeth consisting of ciprofloxacin, metronidazole, and minocycline i.e., TAP in group A and modified combinations consisting of ciprofloxacin, ornidazole, and minocycline in group B. Where they found both the groups showed significant results. However, group B had better result than group A clinically and radiographically. ^14^

A similar study was conducted in year 2013 by Sabrah *et al.* comparing the antibacterial activity of Triple Antibiotic Paste (TAP) and Double Antibiotic Paste (DAP) against *Enterococcus faecalis and Porphyromonas gingivalis* in comparison to calcium hydroxide. They found that both TAP and DAP were significantly more efficient in the reduction of bacterial biofilms in comparison to calcium hydroxide. ^15^

Similarly a study conducted by Dahake P *et al* (2020) on Minimum Inhibitory Concentration (MIC) and Minimum Bactericidal Concentration (MBC) of Clindamycin (C), Metronidazole (M), Doxycycline (D) as well as their combination CMD that clindamycin could inhibit the levels of *C albicans* by 90% but Metronidazole could not inhibit the *C. albicans* and also concluded that combination CMD can be used to achieve predictable better results. ^16^

Govindaraju *et al* (2021) had evaluated Antibiotic sensitivity of the following intracanal medicaments grouped into 4 groups consisting of TAP (Ciprofloxacin, metronidazole, and doxycycline); Calcium hydroxide paste; Odontopaste and Sterile saline (0.9%) as a negative control against *E. Faecalis, S. mutants, and S. aureus.* In which they found that TAP showed the highest inhibition zone to conclude that TAP had better antibacterial activity than other groups. ^17^

However, Minocycline causes discoloration of tooth as stated by various authors. ^11^^,^^12^ For the same reason, Sara Alsubait *et al* (2020) in their study had replaced minocycline with Cefaclor. ^11^

Considering the disadvantages associated with conventional Triple Antibiotic Paste and reduced antimicrobial efficacy of Metronidazole, a new formulation in the form of 3C paste consisting of Ciprofloxacin, Clindamycin and Cefaclor will be prepared for its use as a root canal filling material for primary teeth.

## Data Availability

No data are associated with this article.
