# Supplementary material for: Comparative evaluation of antimicrobial efficacy of 3C antibiotic paste with triple antibiotic paste as root canal filling material for primary teeth: An in-vitro study
Source: F1000Res. 2024 Jan 11;12:754. Originally published 2023 Jun 27. [Version 2] doi: 10.12688/f1000research.133458.2 (PMC11962363; doi:10.12688/f1000research.133458.2)
Supplement: Peer review history

# Reviewer report: Reviewer response for version 2

**DOI:** 10.5256/f1000research.161170.r249916

**Published:** 2025-04-01

**Authors:** Mohammad Adhitya Latief

**Affiliations:** 1 University of Indonesia, Depok, West Java, Indonesia

**Strong point and advantage of this research are :**

 1. Novel Formulation: The study introduces a new antibiotic paste (3C paste) consisting of Ciprofloxacin, Clindamycin, and Cefaclor, which aims to improve antimicrobial efficacy against resistant microorganisms like E. faecalis and C. albicans in primary teeth. ​

 This formulation potentially reducing aesthetic concerns associated with root canal treatments. ​

 It has broad-spectrum efficacy which potentially offering a more effective solution for polymicrobial infections in root canals. ​

 2. Potential Clinical Application: If successful, the 3C paste could become a preferred root canal filling material for primary teeth, improving treatment outcomes and patient satisfaction. ​

**Room for Improvement**

 1. Detailed Methodology, regarding the preparation methods for the antibiotic pastes should be elaborated further, including drug specifications, concentrations, and milligram/milliliter details to ensure reproducibility. ​

 2. Long-Term Outcomes, this research should also investigate the long-term outcomes of using the 3C paste, including its impact on tooth integrity, patient comfort, and recurrence of infections especially if this could be done in in vitro studies.

Is the study design appropriate for the research question?

Yes

Is the rationale for, and objectives of, the study clearly described?

Yes

Are sufficient details of the methods provided to allow replication by others?

Yes

Are the datasets clearly presented in a useable and accessible format?

Yes

Reviewer Expertise:

Dentoalveolar Surgery, oral and maxillofacial trauma, dental and maxillofacial implants

I confirm that I have read this submission and believe that I have an appropriate level of expertise to confirm that it is of an acceptable scientific standard.

# Reviewer report: Reviewer response for version 2

**DOI:** 10.5256/f1000research.161170.r249912

**Published:** 2024-06-19

**Authors:** Srinidhi S R

**Affiliations:** 1 Dr. D. Y. Patil Dental College & Hospital, Pune, India

Introduction -

 The study synopsis gives a background about the type of microbes and their persistence in root canals of deciduous teeth. The common microbes which are present even after routine canal cleaning and disinfection methods have been mentioned. However, I would like to put a few additions the authors can add which will help the readers in better understanding the rationale for the use of antibiotic pastes.

 Both of these suggestions are **minor revisions**. The results of this study will give a better idea for the clinician as to the protocol to be applied in such recalcitrant cases.

 1. The authors have mentioned about the persistence of *C albicans *and *E faecalis*. It can also be added that in spite of various irrigants and activation methods (give examples), these microbes resist removal.

 2. Mention any study which has used Clindamycin as one of the medicaments. The reference given is of the textbook only.

Is the study design appropriate for the research question?

Yes

Is the rationale for, and objectives of, the study clearly described?

Yes

Are sufficient details of the methods provided to allow replication by others?

Yes

Are the datasets clearly presented in a useable and accessible format?

Not applicable

Reviewer Expertise:

Endodontic microbiology, irrigants and techniques

I confirm that I have read this submission and believe that I have an appropriate level of expertise to confirm that it is of an acceptable scientific standard, however I have significant reservations, as outlined above.

# Reviewer report: Reviewer response for version 1

**DOI:** 10.5256/f1000research.146449.r184034

**Published:** 2023-08-22

**Authors:** Shiva Shojaeian

**Affiliations:** 1 Department of Endodontics, School of Dentistry, Shahid Beheshti University of Medical Sciences, Tehran, Iran

The study would be improved if it is conducted to evaluate the antimicrobial efficacy of 3C paste and triple antibiotic on *E. faecalis* and *C. albicans* biofilm. Microbial biofilms in the root canal, penetrate into the dentinal tubules which are more challenging to eradicate compared with microorganisms growing in the cultivation media. *E. faecalis* and *C. albicans can cultivate in root canal specimens to form microbial biofilm on dentin therefore the condition is more similar to the clinical situation*

 The methods of antibiotic preparation should be elaborated thoroughly including Drug specifications, concentration, and milligram/milliliter.

 The specimens should be assigned to the experimental and negative and positive control groups.

Is the study design appropriate for the research question?

Partly

Is the rationale for, and objectives of, the study clearly described?

Yes

Are sufficient details of the methods provided to allow replication by others?

No

Are the datasets clearly presented in a useable and accessible format?

No

Reviewer Expertise:

Endodontic regenersation

I confirm that I have read this submission and believe that I have an appropriate level of expertise to confirm that it is of an acceptable scientific standard, however I have significant reservations, as outlined above.

# Response

**Published:** 2023-09-26

**Authors:** Aakriti Chandra

**Affiliations:** Pediatric Dentistry, Sharad Pawar Dental College , Datta Meghe Institute of Higher Education and Research, Wardha, Maharashtra, India

*The study of cultivating the microbial biofilms in root canal will be considered in future studies.*

*the drug specifications will be given in final study once MIC of the dugs has been established.*
